# Pre-Hospital Artificial Intelligence-Guided, Focused Echocardiography in Patients with Acute Chest Pain for Diagnosis of Acute Coronary Syndrome

**DOI:** 10.3390/jcm14227938

**Published:** 2025-11-09

**Authors:** Soufiane El Kadi, Mark Zanstra, Arjen Siegers, Berto J. Bouma, Albert C. van Rossum, Otto Kamp

**Affiliations:** 1 Amsterdam Cardiovascular Sciences, Department of Cardiology, Amsterdam UMC, Location VUmc, 1081 HV Amsterdam, The Netherlands; ac.vrossum@amsterdamumc.nl; 2 Ambulance Amsterdam, Zaanstreek-Waterland, 1502 DR Zaandam, The Netherlands; m.zanstra@ambulanceamsterdam.nl (M.Z.); asiegers@ambulanceamsterdam.nl (A.S.); 3 Amsterdam Cardiovascular Sciences, Department of Cardiology, Amsterdam UMC, Location AMC, 1105 AZ Amsterdam, The Netherlands; b.bouma@amsterdamumc.nl

**Keywords:** pre-hospital, artificial intelligence, echocardiography, ACS, longitudinal strain

## Abstract

**Background:** Acute chest pain is a common emergency with only 10–20% of cases attributable to acute coronary syndrome (ACS). Rapid and accurate pre-hospital diagnosis remains challenging, particularly for non-ST elevation ACS, where ECG findings may be inconclusive. AI-guided focused cardiac ultrasound (FoCUS) using handheld devices offers a potential solution by enabling immediate functional cardiac assessment. The aim was to investigate the feasibility and diagnostic performance of pre-hospital AI-guided FoCUS for detecting ACS in patients with acute chest pain. **Methods:** In this single-center, prospective pilot study, 75 patients with acute chest pain were enrolled. FoCUS examinations were performed by experienced sonographers (72%) and EMS paramedics (28%) using AI-guidance for obtaining the apical 4-chamber (AP4CH), apical 2-chamber (AP2CH), and apical 3-chamber (AP3CH) views. The quality of the obtained images was assessed, and quantitative measurements—including left ventricular ejection fraction (LVEF) and global longitudinal strain (GLS)—were analyzed. Diagnostic performance was subsequently evaluated using ROC curve analysis. **Results:** At least one apical view was acquired in 91% of patients, with sonographer achieving higher acquisition rates than paramedics (96% vs. 67% for the AP4CH view). Complete acquisition of all apical views was achieved in 67% of cases (83% vs. 24%), and image quality was high across views, with median scores ranging from 83% to 100%. GLS yielded an AUC of 0.76 (89% sensitivity, 56% specificity) and LVEF yielded an AUC of 0.65 (75% sensitivity, 73% specificity). In patients with intermediate to high HEAR-scores (>3), lower LS-AP4CH values were associated with ACS. **Conclusion:** Pre-hospital AI-guided FoCUS is feasible and shows promise for ACS detection, although quantitative parameters do not yet outperform established clinical scores. Enhanced training and further refinement of AI algorithms are needed before widespread implementation.

## 1. Introduction

Acute chest pain forms a major reason for visits to the emergency department and is expected to increase in frequency due to an aging population [1,2]. Although prompt management is warranted in patients with acute chest pain, only 10–20% of these patients actually suffer from ACS [3]. Unlike in the case of ST-elevation myocardial infarction (STEMI), non-STEMI (NSTEMI) cannot be diagnosed or ruled out based solely on the electrocardiogram (ECG). Of importance, 25% of NSTEMI patients present with total occlusion of the coronary artery, a feature that has been linked to increased mortality rates [4]. Similarly, diagnosis of unstable angina and myocardial infarction with non-obstructive coronary arteries are complicated by the absence of definitive electrocardiographic markers. Optimizing both diagnosis and risk stratification in the pre-hospital setting could result in improved patient management and efficient transfer of patients to PCI capable centers. Real-time cardiac imaging using focus cardiac ultrasound (FoCUS) provides valuable information on cardiac anatomy and function. FoCUS is characterized by point-of-care cardiac ultrasound aimed at ruling-in or excluding a limited number of severe cardiac pathologies using a short-duration, simplified protocol [5]. Handheld ultrasound devices offer advantages over conventional echocardiography machines in terms of versatility, costs and convenience. Currently, various handheld ultrasound vendors offer artificial-intelligence (AI)-guided acquisition of echocardiography cine loops, enabling novices and briefly trained paramedics to obtain interpretable ultrasound images [6,7]. AI-guided FoCUS in a pre-hospital setting could help identify impaired myocardial contractility as a sign of an ischemic cause of acute chest pain and lead to more effective triage. The aim of this study was to investigate the feasibility of AI-guided FoCUS using handheld ultrasound devices in the ambulance for diagnosis of ACS in patients presenting with acute chest pain.

## 2. Methods

The study was a single-center investigator-initiated, prospective pilot study conducted at the Amsterdam University Medical Center, location VU University Medical Center (Amsterdam, The Netherlands) in collaboration with the Emergency Medical Services (EMS Zaandam, Ambulance Amsterdam, Zaandam the Netherlands). The objective was to assess the feasibility and diagnostic performance of pre-hospital, AI-guided, FoCUS with quantitative analysis of myocardial function to detect ACS in patients presenting with acute chest pain.

### 2.1. Patient Enrollment

For patients with acute chest pain presenting to the emergency medical services, FOCUS was performed by ambulance nurses. Inclusion criteria were acute chest pain, age over 18 years, and decision by the ambulance nurses to transfer to the hospital. Exclusion criteria were previous myocardial infarction, known cardiomyopathy, previous coronary artery bypass surgery, present STEMI, present atrial fibrillation, and shock (defined as systolic blood pressure < 90 mmHg and heart rate > 100 beats per min). The trial was conducted in accordance with the standards for Good Clinical Practice and the Declaration of Helsinki. Ethics approval was obtained by the Institutional Review Board of the Amsterdam University Medical Centers. Oral informed consent was obtained prior to study procedures and deferred written informed consent was provided by all participants.

### 2.2. Standard Care

In compliance with national standards of care protocols for the EMS, patients presenting with acute chest pain were examined clinically: localization and severity of chest pain were documented, vital parameters were obtained, and a 12-lead ECG was acquired. If ACS was suspected, a loading dose of 180 mg acetyl salicylic acid was administered prior to hospitalization. The History, ECG, Age, and Risk factors (HEAR) score was determined pre-hospital, while the History, ECG, Age, Risk factors, and Troponin (HEART) score was calculated after troponin testing in the hospital. The final diagnosis of ACS was established using the criteria outlined in the 2023 European Society of Cardiology guidelines for acute coronary syndromes [8].

### 2.3. Equipment and Training

Six ambulances were equipped with a Kosmos Bridge handheld ultrasound device (EchoNous, Inc., Redmond, WA, USA) with an attached 2–5 MHz phased-array transducer. The Kosmos ultrasound device provides AI-powered guiding for fanning, rotating, and repositioning the ultrasound probe to acquire optimal-quality images. Concurrently, the system delivered immediate image grading to assist the nurse operator in achieving optimal probe placement. The quality assessment algorithm of the EchoNous device was developed using proprietary training data. Prior to study inclusion, all EMS paramedics from EMS Zaandam completed a structured one-day echocardiography training consisting of both theoretical instruction and practical hands-on sessions. The training covered basic ultrasound principles, cardiac anatomy, and acquisition of standard apical views. By the end of the program, participants were familiarized with the Kosmos ultrasound device and demonstrated competency in acquiring apical 4-chamber (AP4CH), apical 2-chamber (AP2CH), and apical 3-chamber (AP3CH) views with AI support. Competency was assessed by experienced sonographers who verified image adequacy before study participation. An experienced sonographer participated in patient inclusion and ultrasound examination. EMS personnel were instructed to perform FoCUS within 10 min; if the examination could not be completed within this timeframe, it was to be aborted. The exact timing of each scan was not recorded.

### 2.4. Image Analysis

All images were digitally stored and analyzed using vendor-independent, proprietary software (Image Arena 4.6, TomTec Imaging Systems, Unterschleissen, Germany). The quality of the individual myocardial segments for each apical view was assessed using a 3-point scale: 100% corresponding to good quality, a score of 50% corresponding to moderate quality and a score of 0% corresponding to poor quality. The average quality for each myocardial segment was calculated by summing the scores for all patients associated with that segment and then dividing the total by the number of patients. Additionally, a total of 6 myocardial segments were evaluated for each apical view (18-segments in total), and the average score of these 6 segments was calculated to determine the quality percentage per apical view per examination. Subsequently, a median score was calculated per apical view. All quality analyses were performed by a blinded, experienced imaging cardiologist. Left ventricular ejection fraction (LVEF, %) was measured using Simson’s Biplane method. The software provided end-systolic global longitudinal strain (GLS, %) measurements using 2D speckle tracking. Manual adjustment of the endocardial border was performed when necessary to ensure accurate tracking. Additionally, longitudinal strain of the apical 4-chamber (LS-AP4CH), apical 2-chamber (LS-AP2CH), apical 3-chamber (LS-AP3CH), ejection fraction based on apical 4-chamber view (EF-AP4CH) and based on apical 2-chamber view (EF-AP2CH) were provided.

### 2.5. Statistical Analysis

Continuous variables were expressed as mean and standard deviation or median and interquartile range, while categorical variables were represented as number and percentages. Independent-samples *t*-tests and Mann–Whitney *U* test were used to compare continuous data between groups for normally and non-normally distributed variables, respectively. Receiver operator characteristic curve analysis, along with the Youden index, was utilized to determine optimal cut-off values. Sensitivity, specificity, negative and positive predictive values and diagnostic accuracy were reported as percentages with the corresponding 95% confidence intervals. Statistical significance was defined as a *p*-value less than 0.05. ROC curve analysis was performed using MedCalc Statistical Software version 20.0.0 (MedCalc Software Ltd., Ostend, Belgium), all other statistical analyses were performed using SPSS (version 28, International Business Machines Inc., Armonk, NY, USA).

## 3. Results

**Figure 1:**
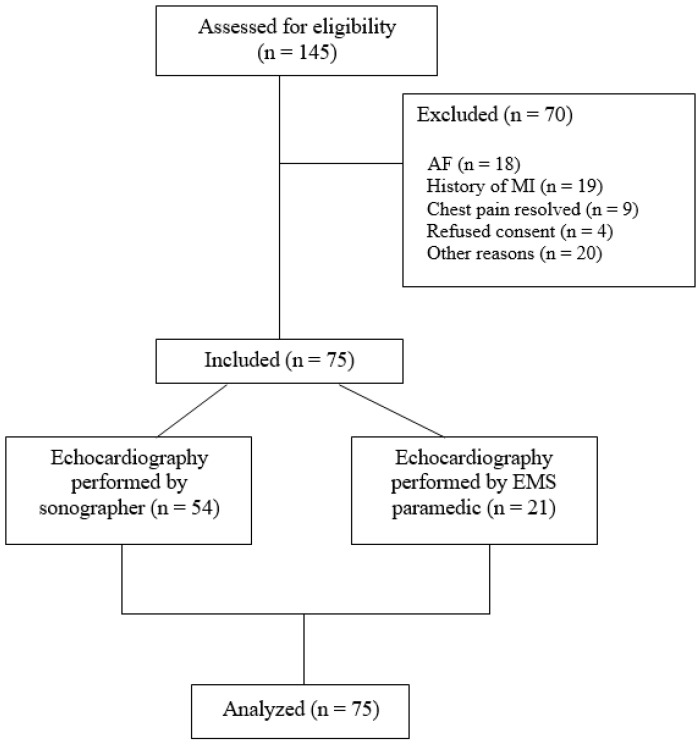
Enrollment diagram. Abbreviations: AF = atrial fibrillation; EMS: emergency medical services; MI: myocardial infarction.

Between April 2024 and December 2024, 145 patients with acute chest pain were screened. After exclusion of 70 patients, a total of 75 patients were included (Figure 1). FoCUS echocardiography was performed by an experienced sonographer in 54 (72%) patients and by EMS paramedics in 21 patients (28%). Mean age was 61 (±12) years and 48% of patients were male (Table 1). Diabetes mellitus was present in 10 (13%) patients, while 37 (49%) patients had hypertension. Four (5%) patients had a history of obstructive coronary artery disease. Median HEAR, which reflects history, ECG, age and, risk factors for atherosclerotic disease was 4 (interquartile range [IQR]: 3–5). In total, 31 (41%) patients underwent at least one form of imaging during index presentation: chest X-ray in 27% of all patients, transthoracic echocardiography in 19% and CT-scan in 3%. Coronary angiography was performed in 15% of patients followed by PCI in 11% of all patients. Culprit vessel was left anterior descending artery in six patients (75% of ACS) and left circumflex artery in two patients (25%). Proximal lesions were observed in three patients (38%), mid lesions in four (50%) and distal lesions in one (12%).

**Figure 2:**
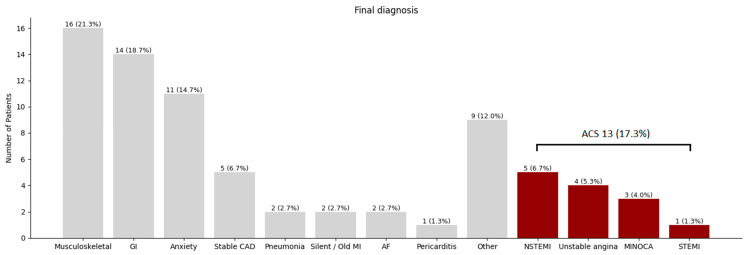
Frequency of final diagnoses. Abbreviations: ACS = acute coronary syndrome; AF = atrial fibrillation; CAD = coronary artery disease; GI = gastro-intestinal; MI = myocardial infarction; MINOCA = myocardial infarction with no obstructive coronary arteries; NSTEMI = non ST-elevation myocardial infarction.

Figure 2 provides information on the frequency of final diagnosis in this patient cohort. In the majority of patients, musculoskeletal causes (21%), gastro-intestinal causes (19%) and anxiety (15%) were determined to be the source of chest pain. Five (7%) patients had stable coronary artery disease, and two (3%) patients had a silent or undiagnosed older myocardial infarction. Acute coronary syndrome was diagnosed in 13 (17%) patients.

### 3.1. Image Acquisition

At least one apical view was successfully acquired in 91% of patients overall, with a markedly higher success rate among sonographers (96%) compared to paramedics (76%) (Table 2). Acquisition of two apical views was accomplished in 83% of patients, while complete acquisition of all apical views was achieved in 67% of cases, again with higher rates among sonographers (68% vs. 24%). The AP4CH view was obtained in 88% of patients overall, with acquisition rates of 96% for sonographers and 67% for paramedics. The AP2CH and AP3CH views were acquired in 73% and 79% of patients, respectively, with sonographers recording higher acquisition rates (85% and 93%) compared to paramedics (both 43%).

### 3.2. Image Quality

**Figure 3:**
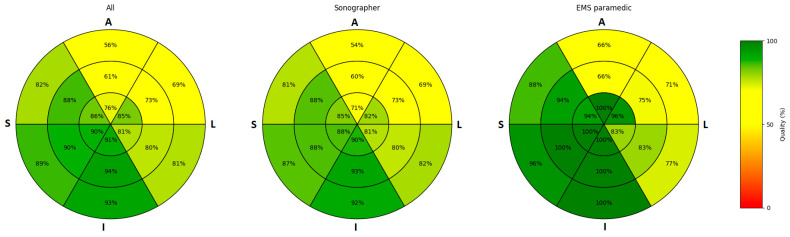
Average image quality per myocardial segment. Abbreviations: A = anterior; L = lateral; I = inferior; S = septal; EMS: emergency medical services.

Regarding image quality, the AP4CH view demonstrated a median quality of 96% (IQR: 83–100%) overall, with sonographers and paramedics achieving median values of 96% (IQR: 75–100%) and 92% (IQR: 83–100%), respectively. For the AP2CH view, the overall median quality was 83% (IQR: 71–100%), with similar performance among sonographers and a notably higher median quality of 100% (IQR: 83–100%) among paramedics. The AP3CH view exhibited a median quality of 100% (IQR: 75–100%) overall, with sonographers matching this value and paramedics reaching a median quality of 92% (IQR: 75–100%). Figure 3 displays the average quality for each segment across all ultrasound acquisitions, with separate breakdowns for sonographer and EMS paramedics.

### 3.3. Diagnostic Performance

**Figure 4:**
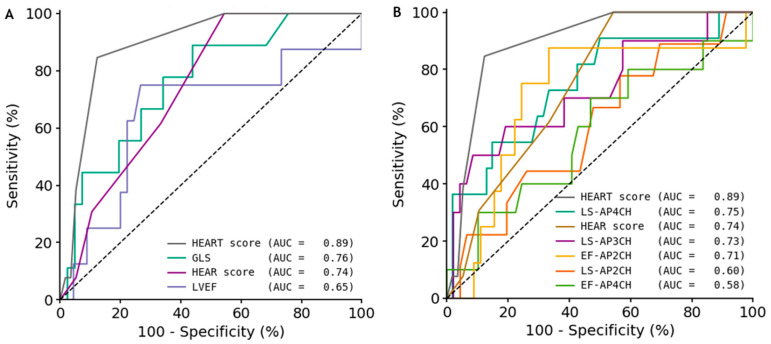
ROC curves for quantitative measures of myocardial function. (**A**): ROC curves for global measures of left ventricular function and HEAR-score, compared to HEART-score. (**B**): ROC curves for regional measures of left ventricular function requiring one apical view only, and HEAR-score, compared to HEART-score. The AUC values indicate overall accuracy. Abbreviations: AUC = area under the curve; EF-AP4CH = ejection fraction-apical 4-chamber; EF-AP2CH = ejection fraction-apical 2-chamber; HEAR(T) = history, ECG, age, risk factors (and troponin); LS-AP4CH = longitudinal strain-apical 4-chamber; LS-AP2CH = longitudinal strain-apical 2-chamber; LS-AP3CH = longitudinal strain-apical 3-chamber; ROC = receiver operating curve.

Compared to the HEART-score (Area Under the Curve [AUC] 0.89), GLS and LVEF yielded lower diagnostic accuracy, with AUCs of 0.76 and 0.65 and optimal cut-off values of −17.5% and 48.5%, respectively (Figure 4). GLS demonstrated high sensitivity (89%) but modest specificity (56%), whereas LVEF provided a more balanced performance with 75% sensitivity and 73% specificity (Table 3). Individual LS and EF parameters varied in performance: LS-AP4CH achieved an AUC of 0.75 with high sensitivity (91%) yet limited specificity (50%), while LS-AP3CH demonstrated excellent specificity (91%) and overall accuracy (84%), despite lower sensitivity (50%). Among EF parameters, EF-AP2CH showed a more balanced profile (AUC 0.71, 88% sensitivity, 67% specificity, and 70% accuracy), whereas EF-AP4CH performed less favorably. The HEAR-score-reflecting pre-hospital clinical examination and ECG-had an AUC of 0.74, 100% sensitivity, but only 46% specificity.

### 3.4. Intermediate–High Risk

**Figure 5:**
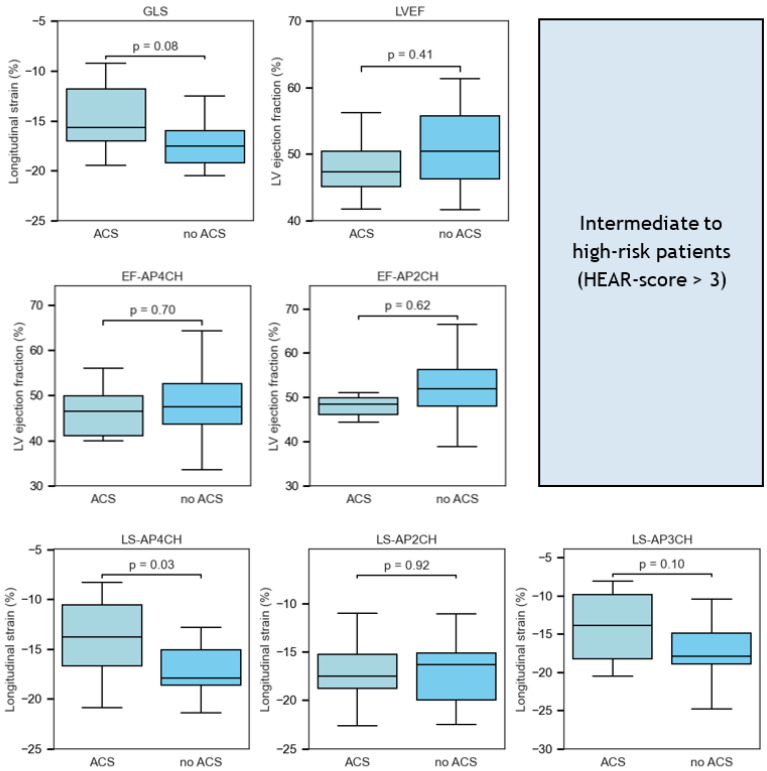
Figure. Box-and-whisker plots of quantitative measures of systolic left ventricular function comparing intermediate- to high-risk patients (HEAR-score > 3) with or without acute coronary syndrome (ACS). Median values, interquartile ranges, and *p*-values are displayed for each parameter. Note that LS-AP4CH showed statistically significant differences between the ACS and no-ACS groups. Abbreviations: ACS = acute coronary syndrome; AUC = area under the curve; EF-AP4CH = ejection fraction-apical 4-chamber; EF-AP2CH = ejection fraction-apical 2-chamber; HEAR = history, ECG, age, risk factors; LS-AP4CH = longitudinal strain-apical 4-chamber; LS-AP2CH = longitudinal strain-apical 2-chamber; LS-AP3CH = longitudinal strain-apical 3-chamber; ROC = receiver operating curve.

In patients with an intermediate to high HEAR-score (>3), LS-AP4CH was lower in patients with ACS compared to patients without ACS (−13.7 ± 3.2 vs. −17.2 ± 3.6, *p* = 0.04, Figure 5). LS-AP3CH (−13.9 [−18.3–−9.9] vs. −18.0 [−18.9–−14.9], *p* = 0.08) and GLS (−15.7 [−17.1–−11.8] vs. −17.6 [−19.2–−16.0], *p* = 0.08) tended to be lower in patients with ACS in comparison with patients without ACS. All other strain and ejection fraction variables were not statistically significantly different.

## 4. Discussion

This pilot study demonstrates that pre-hospital AI-guided FoCUS is a feasible approach for the evaluation of patients presenting with acute chest pain. Our findings indicate that, with appropriate training, both an experienced sonographer and EMS paramedics can acquire diagnostic-quality apical views, although successful image acquisition was achieved more frequently when FoCUS was performed by a trained sonographer. We observed an overall high success rate in obtaining at least one apical view, with sonographers achieving higher acquisition rates compared to paramedics. In particular, the AP4CH view was most consistently obtained (88% overall), corroborating previous reports that emphasize the relative ease of acquiring this view even among ultrasound novices [7]. Notably, earlier studies of handheld echocardiography performed by novice users have reported over 70% interpretable images, albeit following substantially longer training durations than the one-day training provided in our trial [9,10]. The lower rate of complete image acquisition among paramedics limits the feasibility of quantitative assessments, as evidenced by the limited number of patients with successful GLS and LVEF measurements. This observation underscores the need for more comprehensive training to enhance image acquisition proficiency.

To our knowledge, this is the first clinical investigation to evaluate the diagnostic performance of quantitative measures of LV function using ultrasound images acquired in the pre-hospital acute setting. As a standalone measure, GLS demonstrates moderate diagnostic value with an AUC of 0.76 for detecting ACS, outperforming LVEF which shows only fair performance (AUC 0.65). GLS also offers higher sensitivity compared to LVEF, albeit at the cost of lower specificity. Notably, LS-AP4CH produced an AUC comparable to that of GLS, indicating that this single view measurement may be sufficient to detect or exclude ACS. This finding is particularly compelling given that the AP4CH view was successfully obtained in the majority of patients.

Originally developed to predict the risk of major adverse cardiac events, the HEART score categorizes scores of three or less as corresponding to a one to three percent risk of adverse outcomes [11,12]. More recent evidence suggests that the HEAR score, which omits troponin assessment, can reliably exclude acute myocardial infarction when stringent threshold values are applied [13,14]. In this study, the HEAR score proved effective in ruling out ACS, as no cases were observed in patients with a score of 4 or lower. In patients with intermediate to high HEAR scores, significant differences in LS-AP4CH were observed between patients with and without ACS, indicating that this parameter may reliably be used as differentiation in this subgroup. Additionally, both GLS and LS-AP3CH differences showed trends toward statistical significance. Previous studies on pre-hospital echocardiography for chest pain relied primarily on the presence of wall motion abnormalities for the detection of ACS [15,16]. In contrast, our approach extends these findings by employing quantitative ejection fraction and strain analysis, which may detect subtle myocardial dysfunction earlier than conventional wall motion assessment. This method, particularly when automated via AI-guidance, also has the advantage of reducing operator- and reader-dependency and ensuring reproducibility of measurements [17,18].

Patients presenting with acute chest pain in conjunction with impaired GLS or LVEF—but without elevated cardiac biomarkers—represent a particularly challenging diagnostic subset. On one hand, while the absence of increased troponin levels per ESC criteria may effectively rule out NSTEMI, acute chest pain in these patients might still result from a relative oxygen supply-demand mismatch in the setting of a critical coronary stenosis that has not yet led to measurable myocardial injury. On the other hand, these abnormal findings may reflect chronic ischemic cardiomyopathy, with or without acute exacerbation due to further narrowing of an existing stenotic lesion. As further ischemia testing was not performed routinely in our study, it remains unclear whether the reduced functional ultrasound parameters represent true positive findings of an acute process or are simply markers of chronic remodeling.

In addition to its potential role in ACS detection, FoCUS may also complement point-of-care troponin assays. The recent ARTICA study demonstrated utility of point-of-care troponin in safely ruling out ACS in patients with low HEAR-score [19]. However, its effectiveness may be limited in early presenters where biomarker levels remain below the diagnostic threshold. Compared with current point-of-care assays, high-sensitivity troponin offers markedly improved analytical sensitivity, enabling detection of lower troponin concentrations and earlier identification of myocardial injury, which may further strengthen early ACS rule-out strategies in the pre-hospital setting. Conversely, although elevated troponin levels are commonly associated with myocardial injury, they can also be seen in non-cardiac conditions including pulmonary embolism, sepsis, or renal dysfunction. In such scenarios, AI-guided FoCUS can provide immediate structural and functional cardiac insights that, when combined with biochemical data, may enhance overall diagnostic accuracy and patient triage.

Beyond the assessment of ACS, pre-hospital FoCUS may have broader applications in acute cardiovascular care. For instance, rapid evaluation of left ventricular function, valvular abnormalities, or pericardial effusion in patients with suspected heart failure or other non-ACS conditions could facilitate timely therapeutic decisions. Moreover, in subpopulations where clinical presentations are atypical, the use of FoCUS might refine risk stratification and support more targeted interventions.

Expanding on the potential impact of AI-guided FoCUS, the implementation of pre-hospital applications involves important economic and logistical considerations, including device procurement costs, structured training sessions for EMS personnel, and the need for supervision or validation by experienced operators. While our pilot study demonstrates feasibility, scaling this approach across emergency services may require careful evaluation of these resource demands. More broadly, AI applications in cardiology are rapidly expanding, including automated imaging interpretation and reporting, risk stratification, personalized interventions, and AI-assisted patient support and education. Effective integration of AI into pre-hospital and in-hospital cardiac care, particularly through collaboration between clinicians and AI developers, could enhance efficiency, reproducibility, and diagnostic accuracy, highlighting its broader translational potential [20].

### Limitations

Several limitations warrant consideration. First, the single-center design and modest sample size limit the generalizability of our findings. Second, the study population was selected by excluding patients with prior myocardial infarction, cardiomyopathy, significant arrhythmias, and shock, which may not reflect the full spectrum of patients encountered in pre-hospital care. Third, although the AI-guided ultrasound device facilitated image acquisition, operator dependency remained evident, as indicated by the lower performance among paramedics compared to sonographers. Fourth, all analyses were conducted offline rather than in real-time during image acquisition. Finally, the brief training provided to EMS personnel may not have been sufficient to achieve optimal proficiency in FoCUS, suggesting that extended training protocols could further enhance diagnostic accuracy. Refining AI algorithms to improve image acquisition by ultrasound novices may help develop FoCUS into a more comprehensive prehospital diagnostic tool.

## 5. Conclusions

In summary, our study provides preliminary evidence that prehospital AI-guided FoCUS is a feasible method for the evaluation of acute chest pain. Although quantitative echocardiographic parameters show promise, their diagnostic performance currently does not surpass that of established clinical scoring systems such as the HEART score. These findings highlight the need for further research to optimize both training and technology before widespread implementation can be recommended.

## Figures and Tables

**Table 1:** Clinical characteristics.

	*n* = 75
Age, years	61 (±12)
Male sex, *n* (%)	36 (48)
Body mass index, kg/m^2^	29 (25–31)
Diabetes mellitus, *n* (%)	10 (13)
Hypertension, *n* (%)	37 (49)
Dyslipidemia, *n* (%)	26 (35)
Smoking, *n* (%)	20 (27)
Positive family history, *n* (%)	20 (27)
History of atherosclerotic disease, *n* (%)	10 (13)
History of obstructive CAD, *n* (%)	4 (5)
History of PCI, *n* (%)	3 (4)
Heart rate, min^−1^	77 (71–98)
Systolic blood pressure, mmHg	159 (145–183)
Diastolic blood pressure, mmHg	95 (83–100)
Oxygen saturation, %	98 (97–99)
HEAR-score	4 (3–5)
HEART-score	4 (3–6)
Imaging performed, *n* (%)	31 (41)
Chest X-ray, *n* (%)	20 (27)
TTE, *n* (%)	14 (19)
CT-a scan, *n* (%)	2 (3)
CAG in non PCI-capable center	4 (5)
CAG in PCI-capable center	11 (15)
ACS	13 (17)
PCI performed	8 (11)

Abbreviations: ACS = acute coronary syndrome; CAD = coronary artery disease; CAG = coronary angiography; CT = computed tomography; HEAR-score: History, ECG, Age and Risk factors; HEART-score = History, ECG, Age, Risk factors, and Troponin; PC = percutaneous coronary intervention; TTE = transthoracic echocardiography.

**Table 2:** Ultrasound acquisition, quality and measurements.

	Total (*n* = 75)	Sonographer (*n* = 54)	Paramedic (*n* = 21)
Acquisition			
One apical view acquired, *n* (%)	68 (91)	52 (96)	16 (76)
Two apical views acquired, *n* (%)	62 (83)	51 (94)	11 (52)
All apical views acquired, *n* (%)	50 (67)	45 (83)	5 (24)
AP4CH view acquired, *n* (%)	66 (88)	52 (96)	14 (67)
AP2CH view acquired, *n* (%)	55 (73)	46 (85)	9 (43)
AP3CH view acquired, *n* (%)	59 (79)	50 (93)	9 (43)
Quality			
AP4CH view %, median (IQR)	96 (83–100)	96 (75–100)	92 (83–100)
AP2CH view %, median (IQR)	83 (71–100)	83 (69–100)	100 (83–100)
AP3CH view %, median (IQR)	100 (75–100)	100 (75–100)	92 (75–100)
Successful measurements			
GLS, *n* (%)	50 (67)	45 (83)	5 (24)
LVEF, *n* (%)	53 (71)	44 (82)	9 (43)

Abbreviations: AP4CH = apical 4-chamber; AP2CH = apical 2-chamber; AP3CH = apical 3-chamber; GLS = global longitudinal strain; LVEF = left ventricular ejection fraction.

**Table 3:** Diagnostic performance of strain and ejection fraction measures compared to HEAR, HEART-score to detect ACS.

Predictor	AUC (95% CI)	Threshold	Sens (95% CI)	Spec (95% CI)	PPV (95% CI)	NPV (95% CI)	Acc (95% CI)
**HEART-score**	0.89 (0.80–0.97)	6.0	0.85 (0.60–1.00)	0.88 (0.78–0.95)	0.61 (0.38–0.84)	0.96 (0.90–1.00)	0.87 (0.79–0.94)
**GLS**	0.76 (0.58–0.92)	−17.5	0.89 (0.64–1.00)	0.56 (0.40–0.71)	0.31 (0.14–0.48)	0.96 (0.86–1.00)	0.62 (0.50–0.76)
**LS-AP4CH**	0.75 (0.55–0.90)	−17.5	0.91 (0.69–1.00)	0.50 (0.37–0.64)	0.27 (0.14–0.41)	0.96 (0.89–1.00)	0.57 (0.45–0.69)
**HEAR-score**	0.74 (0.62–0.86)	4.0	1.00 (1.00–1.00)	0.46 (0.33–0.59)	0.30 (0.15–0.45)	1.00 (1.00–1.00)	0.56 (0.44–0.67)
**LS-AP3CH**	0.73 (0.52–0.91)	−13.0	0.50 (0.17–0.83)	0.91 (0.83–0.98)	0.56 (0.22–0.88)	0.90 (0.80–0.98)	0.84 (0.74–0.93)
**EF-AP2CH**	0.71 (0.47–0.90)	51.0	0.88 (0.57–1.00)	0.67 (0.52–0.80)	0.32 (0.14–0.53)	0.97 (0.89–1.00)	0.70 (0.58–0.81)
**LVEF**	0.65 (0.37–0.89)	48.5	0.75 (0.40–1.00)	0.73 (0.59–0.85)	0.33 (0.12–0.56)	0.94 (0.86–1.00)	0.74 (0.62–0.85)
**LS-AP2CH**	0.60 (0.40–0.79)	−18.8	0.78 (0.50–1.00)	0.43 (0.29–0.58)	0.21 (0.09–0.35)	0.91 (0.77–1.00)	0.49 (0.36–0.62)
**EF-AP4CH**	0.58 (0.37–0.79)	47.7	0.70 (0.40–1.00)	0.53 (0.39–0.67)	0.23 (0.09–0.40)	0.90 (0.78–1.00)	0.56 (0.42–0.68)

Abbreviations: ACS = acute coronary syndrome; AUC = area under the curve; CI = confidence interval; Sens = sensitivity; Spec = specificity; PPV = positive predictive value; NPV = negative predictive value; Acc = accuracy; GLS = global longitudinal strain; LS-AP4CH = longitudinal strain in apical 4-chamber view; LS-AP3CH = longitudinal strain in apical 3-chamber view; LS-AP2CH = longitudinal strain in apical 2-chamber view; EF-AP2CH = ejection fraction in apical 2-chamber view; EF-AP4CH = ejection fraction in apical 4-chamber view; LVEF = left ventricular ejection fraction; HEAR-score = history, ECG, age, risk factors score; HEART-score = history, ECG, age, risk factors, troponin score.

## Data Availability

The data presented in this study are available from the corresponding author upon reasonable request.

## Abbreviations

ACS	acute coronary syndrome
AI	artificial intelligence
AP4CH	apical 4-chamber
AP2CH	apical 2-chamber
AP3CH	apical 3-chamber
AUC	area under the curve
CAG	coronary angiography
ECG	electrocardiogram
EF	ejection fraction
FoCUS	focus cardiac ultrasound
GLS	global longitudinal strain
HEAR(T)	history, ECG, age, risk factors (troponin)
LV	left ventricle
LS	longitudinal strain
LVEF	left ventricular ejection fraction
NSTEMI	non ST-elevation myocardial infarction
PCI	percutaneous coronary intervention
STEMI	ST-elevation myocardial infarction
